# Faculty recruitment to academic health systems in difficult times: defining current challenges and best practices

**DOI:** 10.1016/j.acpath.2026.100246

**Published:** 2026-04-06

**Authors:** David N. Bailey, Shelli Herman, Jan D. Hirsch

**Affiliations:** a Department of Pathology, University of California San Diego, La Jolla, CA, USA; b Shelli Herman and Associates, Inc., Los Angeles, CA, USA; c Department of Clinical Pharmacy Practice, University of California Irvine, Irvine, CA, USA

**Keywords:** Evaluation, Finances, Interviews, Search, Selection

## Abstract

Faculty recruitment to academic health systems (AHS) is difficult even in the best of times due to the inherently complex interfaces between health sciences schools, medical centers, affiliated institutions, faculty, and administration as well as the limited availability of top talent. However, with diminishing research and healthcare funding, coupled with proliferating regulations, recruitment has become even more challenging. These factors make it more important than ever to define best practices in AHS recruitment and may prompt the recruitment of internal candidates rather than external ones, the pursuit of philanthropic support for capacity building and start-up packages, the consolidation of academic units, and the combination of leadership positions. The specific needs will vary considerably depending on the type of position sought (e.g. “rank-and-file” faculty member, department chair, dean, academic medical center chief executive officer) as well as the specific responsibilities expected of them. Leadership roles may be particularly difficult to recruit for at this time and may appear too daunting for some potential candidates to consider. These challenges may lead to a reevaluation of roles and responsibilities, reporting relationships, and, perhaps, the redefinition of the positions themselves. The authors draw on their collective experience to define best practices in recruitment to AHS during these turbulent times.

Although most academic institutions have their own guidelines and policies governing recruitment, there is a paucity of publications on recruitment to academic health systems (AHS), defined as health sciences schools, their affiliated academic medical center(s) (AMC) and faculty practice groups.^1^, ^2^, ^3^, ^4^, ^5^, ^6^, ^7^, ^8^, ^9^, ^10^, ^11^ With increasingly limited budgets, scarce resources, regulatory changes, and intense competition for talent, recruitment to AHS has become especially challenging.^8^^,^^12^, ^13^, ^14^, ^15^, ^16^ Additionally, increasing professional burnout stimulates turnover and complicates recruitment.^17^^,^^18^ Based on their collective experience, the authors wish to share best practices in the AHS academic recruitment process during these unsettling times, recognizing that practices may vary substantially across AHS depending on the stages of development and economic stability of the AHS.

## Types of recruitment

Recruitment to AHS varies by type of position sought, with each having its own set of challenges. The authors summarize here the tiers of positions addressed in this commentary.

### Faculty recruitment

In this era of financial constraints, “rank-and-file” faculty recruitment to AHS is often based on a defined clinical need for the position, with the assumption that the clinical revenue generated will support the position. In such instances, a new position may be created to address increased clinical workload. That said, departments generating the additional work may be the only ones benefiting from increased clinical revenue, while other departments impacted by the “downstream” increased workload may have difficulty in even obtaining authorization to recruit.

The top reasons for turnover have been reported to be professional advancement, salary, personal issues, transition from academia, and retirement.^2^ Finding the right candidate is frequently difficult due to the scarcity of qualified individuals, salary concerns, and local market competition.^2^ Reportedly, the hardest specialty to recruit and retain is adult primary care.^2^ Most faculty recruitments are under the ultimate authority of the academic department chair with some input from the AMC, depending upon the degree of clinical involvement and any financial support provided by the AMC. To the extent that the faculty recruit brings a strong research program, then the department will also need to provide a research start-up package, which may be increasingly difficult in these times of budgetary constraint. As a result, the profile of many academic departments may become increasingly clinical over time.

### Department chairs, institute directors, and center directors

Recruitment of department chairs, institute directors, and center directors is often under the purview of the professional school dean or a higher authority. As such, the process and the recruitment package are usually the obligation of that entity. The individual department, institute, or center is typically only peripherally involved, usually in an advisory capacity. Although institutions are financially constrained during these times, many, if not most, adopt the view that strong leadership is needed during such stressful periods and proceed with such recruitments. Indeed, there are insightful candidates who realize that they can make their mark by leading the academic unit during a crisis. Searches for such leaders should focus on individuals who demonstrate resilience, creativity, and a willingness to lead during difficult times. Also, in the authors' experience, there is increasing emphasis on business skills and strategic thinking in these leadership positions. In fact, these abilities may, in many instances, be as important as, or even more important than, the individual's personal academic portfolio.

### Deans of professional schools

The recruitment of deans of professional schools (e.g. medical, pharmacy, nursing, public health) is typically overseen by a campus official such as a provost, vice chancellor/president, or chancellor/president. As with campus-level institute or center director recruitment, the school is primarily involved in an advisory role, with the final selection made by the responsible campus official and governing body (e.g. regents, board of trustees). Again, strong leadership skills, adaptability, creativity, resilience, strategic thinking, and business skills are especially important during these times.

### Chief executive officer of the affiliated academic medical center

Recruiting the chief executive officer (CEO) of the AMC in the AHS is particularly challenging during times of financial duress as the AMC is often considered to be the financial “engine” that drives the AHS. Most CEO searches seek candidates who are financially savvy, strong leaders, and excellent communicators. Community leaders input is especially important because the AMC serves as a resource for the community and the region in which it resides. Because the AMC interacts with the health sciences professional schools, those entities must also have input. Finally, if those AMCs are owned and/or operated by a university, the input and support of campus leadership (e.g. the president or chancellor) and the university governing body (e.g. board of trustees or regents) are critically important. Because of these complex interactions, recruiting the AMC CEO may be among the most challenging recruitments.

## Process issues and challenges

### Search committee

The significance of the search committee in the hiring process cannot be overstated. Its composition should reflect the constituents the candidate will serve, and the committee size should not be so large as to impede an efficient operation. “Rules of order” must be established by the individual responsible for the hiring (the “hiring authority”), who will typically specify them in the charge to the committee. The charge should explain why the recruitment is taking place and outline the expectations for the position. Institutional policies and guidelines should be articulated including any requirements imposed by the funding source. These should be understood by committee members, and the committee members should be aligned.^3^^,^^4^ It is also important for the hiring authority to establish the scope and any limitations of the search in the charge to the search committee (e.g. national/international search versus internal search, geographic restrictions, salary limitations).

### Advertisement and communication

The position advertisement is exceedingly important as is the development of a communication strategy. Committees have traditionally been responsible for creating and placing ads to achieve a broader reach. Today, a variety of firms specialize in selecting appropriate advertising platforms to maximize exposure and in working with institutions to develop communication plans. These firms skillfully offer solutions tailored to unique needs, enabling a more data-driven approach for both print and online efforts, aimed at building a candidate pool. The advertisement should include the position responsibilities, a description of the entity to be administered by the candidate as well as the institution in which the entity resides, and the background and experience sought from applicants. The application timeline should be clearly articulated, with provision for considering qualified candidates after the initial review deadline has passed if the position has not yet been filled. The advertisement should be sent to the institution's websites, appropriate professional societies, organization listservs, journals, and selected leaders in the field.

### The use of search firms

Using a search firm provides access to a broader network of qualified candidates including passive candidates, and typically saves time, thereby conserving institutional resources. These firms offer specialized expertise, a discreet and confidential process, and a thorough vetting procedure that helps ensure a better cultural fit and long-term retention for critical roles. Firms possess extensive networks of high-performing candidates including those not actively seeking new opportunities. They can also identify candidates with the specific experience, skills, and knowledge required for the role. Reports indicate that the use of search firms also yields more diverse candidate pools.^5^ Especially when a position is difficult to fill, these firms have the experience and access needed to identify and attract the right talent. The selection of the right firm depends on the nature of the position being filled, the contract terms (including the obligation to initiate another search if the chosen candidate leaves the position within a specified period), the availability of staff at the search firm, and the personal interactions between the search committee and the search firm's lead. That said, due to the expense of using search firms, some institutions will rely on their internal recruitment offices and will reserve the use of search firms for higher-level positions.

### Gathering input from stakeholders

It is crucial to gather input from all individuals (“stakeholders”) who will be affected by the outcome of the search. This may include faculty, department chairs, deans, center directors, AMC CEOs, and the community. The search committee can host “listening sessions” with stakeholder groups before launching the search to solicit their views on the position. The committee should carefully organize and consider this input. Even if such input is not ultimately implemented, the fact that it is sought is usually much appreciated by stakeholders.

## Evaluation and selection of candidates

### Internal versus external candidates

It is not uncommon for searches to include internal applicants. These applicants should be evaluated alongside external applicants. Internal candidates have the advantage of usually being able to start the position soon, knowing the organization well and thus not having to “come up to speed,” and not having to relocate, among others. In fact, many successful institutions have formal programs for developing internal leaders.^19^ That said, internal candidates often do not bring a fresh perspective that external candidates do. In contrast, external candidates typically require time to relocate and learn about the institution before engaging with it, and often demand a more comprehensive recruitment package. However, external candidates can bring a new outlook and direction to an institution. Unlike internal candidates, external ones are usually granted a “honeymoon” period to adjust to their new role. In the final analysis, the hiring authority must decide whether the internal or external candidate is a better fit at that time in the institution's history.

### Selection of applicants for interview

After the application timeline closes, the search committee, whether or not assisted by a search firm, must screen applications. The authors have found that asking individual committee members to sort applications into three groups, using the position qualifications described in the advertisement is useful: (1) immediately reject (unqualified); (2) accept for interview (qualified); and (3) hold for backup (acceptable but not top tier) as needed. Then, those applicants whom a majority of the search committee approves for an interview can be invited to a virtual screening interview. It is paramount that the committee maintains the confidentiality of all applicants’ identities.

## The interview process

It is essential that the interview process be standardized to ensure that all applicants are treated equally. This requires preparing a set of questions and assigning them to individual search committee members, who will ask the same question(s) to all applicants during the screening interview, which is usually virtual. All committee members should score each question, preferably on a 1 (worst)–10 (best) scale.

Candidates who receive the highest scores should be invited to a more comprehensive interview, which may be conducted virtually or in-person. Usually, there are no more than three to five such candidates. Again, questions should be standardized and should be more in-depth than those posed during the screening interview. During on-site visits, the candidate should meet with key stakeholders, have one-on-one time with the hiring authority, and receive a tour of the facilities. Ideally, schedules should be consistent in order to ensure a fair and equitable search process.

The authors have found the following “dos” and “don'ts” to be helpful in the search process, whether or not they involve in-person or virtual interviews:

Do

• Recuse yourself from interviewing candidates with whom you might have a conflict of interest.
• Adhere to the appropriate script for questions.
• Be an ambassador for the institution.
• Dress appropriately.
• Maintain candidate confidentiality.

Don't

• Interact with candidates outside of the formal interview process if you are on the search committee.
• Convey to the candidate your opinions about their strengths/weaknesses.
• Talk about the institution's problems unless specifically asked by the candidate.
• Compare candidates against each other or stack rank the pool. Instead, evaluate each candidate on their ability to perform in the position, contribute to organizational growth, and lead in a forward-thinking manner.
• Talk about other candidates.

## Selection of finalist candidates

During the hiring authority's presentation to the committee, it is customary for a request to be made for the committee to recommend a defined number of candidates (often three) to the hiring authority, who will then proceed to negotiate with the candidates. In making the recommendation, the search committee should use both quantitative data (e.g. interview scores) as well as qualitative/subjective data from nonsearch committee interviewers. Some have also used a more holistic approach.^20^

That said, the selection of finalist candidates is often coupled with a perception of the right “fit” for the position's responsibilities and the ability of the candidate to work with both their boss(es) and those reporting to them.^21^

While historically many hiring authorities have asked the search committee to recommend candidates in ranked order, it is increasingly common for the search committee to provide the hiring authority with the relative strengths and weaknesses of the top candidates for the position, allowing the hiring authority to decide with whom to negotiate and in what order. This also avoids the highly awkward situation in which a ranked order becomes known outside the committee.

## Final stages

Once the shortlist of candidates has been presented to the hiring authority, it is incumbent upon that person to begin negotiations with the candidate(s). Depending on the hiring authority's style and the acuity of institutional needs, negotiation may occur with one or more candidates simultaneously. Negotiating with more than one candidate at a time risks leading them to believe each is the top choice, whereas negotiating with only one candidate at a time may prolong the search if that candidate declines an offer. Furthermore, during the negotiation period, the other finalists may lose interest or be recruited elsewhere. Importantly, during negotiations, the hiring authority should be able to assess the candidate's ability to work with them. Ideally, there would be strong consensus on a single candidate, eliminating the need to pursue more than one individual at a time.

Depending on the hiring authority, a reverse site visit can be helpful in selecting the successful candidate for the position. Such visits allow the hiring authority to observe the candidate in their work environment and to understand how they are perceived by their institutional colleagues. Such visits also indicate to the candidate the seriousness of their candidacy. The authors believe that during such visits it is appropriate to provide a preliminary proposal, which, if not accepted by the candidate, absolves the institution from inviting them back for further visits. If the candidate accepts the preliminary proposal, it indicates their seriousness about proceeding to formal negotiations. Implicit in the reverse site visit is that the candidate has shared their candidacy with their home institution. If not, there must be another reason (e.g. “visiting professor”) for the reverse site visit.

Negotiation is as much of an art as a science. It is important to define who will lead it, when it should start, and who will participate. Negotiations should include salary, benefits (including nonfinancial benefits), relocation, spousal/partner considerations, start-up resources, and mentoring opportunities as needed. In rare instances, a shortened work week has been proposed to enhance the package.^22^ A few institutions have even allowed extramural faculty practice with stringent controls.^23^ In assembling the final package, it is often necessary to draw upon multiple resources such as other departments, schools, the dean's office, AMC, and campus support.^6^^,^^7^ Sometimes an “escape clause” or a severance package component is built into the offer to allow departure of the candidate without cause on amicable terms within a defined period.

## What can go wrong?

Searches involve the human element, multiple moving parts, and opportunities for things to go awry. These include, but are not limited to, the following:

• The search committee may^3^^,^^5^^,^^9^
  - Represent too narrow a range of perspectives and interests.
  - Not follow the agreed-upon metrics and standardized process.
  - Allow one or more committee members to dominate the search process.
  - Have stereotype, affinity, or elite-institution biases.
  - Be made too large to satisfy all constituencies, thereby diminishing each member's sense of belonging and contribution. (A large committee is also difficult to convene with 100% participation).
  - Misunderstand or not follow the committee charge.
  - Think of itself as a selection committee instead of a search committee.
  - Breech confidentiality.
• Use of “preselected” targets of opportunity that devalue the search process^3^
• Concerns about relocation, as well as spousal/partner hire needs emerging late in the process^3^
• A search process that is too passive, with the committee assuming that institutional stature will draw the best candidates^9^
• Too slow scheduling of search committee meetings^9^
• Misunderstanding facts and data during negotiation
• Not sufficiently addressing the characteristics, skill sets, and competencies needed by the successful candidate^9^
• For leadership positions, focusing too much on the personal academic portfolio of the candidate and not enough on their ability to lead and inspire teams, to build a financially stable operation, and to communicate a forward-looking strategy^5^
• Not matching candidate expectations with the available package
• Not being sufficiently transparent with applicants
• Not being consistent in messaging
• Not standardizing the search process
• Perception that the search is not being done in earnest due to the presence of a strong internal candidate or a predetermined external candidate
• The search becomes sufficiently protracted that attractive candidates lose interest^9^
• Bad institutional publicity

## Best practices

Generally speaking, recruitment becomes more difficult as the position holds greater authority, owing to the involvement of more stakeholders, which introduces more potential veto points.

The authors have found the following to be best recruitment practices:

• Streamline the committee structure (include a small, core decision-making subgroup).^4^
• Ensure alignment of the committee on the competencies of the ideal candidate.^4^
• Compose the search committee for speed, decision-making, and engagement.^5^
• Consider using a search firm to decrease turnaround time for searches.
• Encourage the search committee to focus on the best talent for the organization's strategy and culture, and not on the number of candidates it can find.^5^
• Compensate beyond salary (e.g. start-up funds, development opportunities, work-life balance).^4^
• Showcase the institution and community culture as a whole.^4^
• Do sufficient preparation work to define the position in outcome terms.^9^
• Consider having an institutional search “ambassador” (one point person whose role is to coordinate senior-level searches and advise the committee on cutting-edge practices).^9^
• Ensure that the search committee is adequately staffed so that it can perform its work thoroughly and efficiently.
• Encourage multiperson (“cluster”) hires with holistic reviews by multidisciplinary committees and consider joint hires between departments.^10^
• At the outset, establish responsibility for recruitment expenses, the role of the search firm, the type of position, and funding for the start-up package.^3^
• Write a position description that encourages a broad candidate pool.^3^
• Consider making the required qualifications minimal and preferred qualifications more broadly defined.^3^
• Emphasize the importance of holistic review in faculty recruitment.^11^
• Encourage search committee members to perform personal outreach to garner qualified applicants.^3^
• Select a search committee chair who can commit to the additional work, has a history of performing successful searches, and is a trusted authority in the institution.^3^
• Involve senior leadership to help lure candidates.^9^
• Perform reverse site visits.^9^
• Use a “high-touch” service (e.g. escorting candidates to interviews, considering needs of spouses/partners, calling candidates who don't continue in the process).^9^
• Use artificial intelligence to filter out unqualified applicants, decrease the time for application review, reduce bias, and offer precise candidate matching.^24^

## Rethinking the process

Although the authors recommend the above-described best practices, they recognize that challenging circumstances may warrant consideration of alternative recruitment approaches. In fact, challenges (and even crises) can create opportunities to change processes that would not be possible otherwise.^25^^,^^26^

These strategies include limiting certain recruitments to internal candidates for a specified period; seeking philanthropic sources for start-up packages; consolidating multiple leadership roles into a single role to boost efficiency, reduce costs, and make the position more attractive; and, perhaps, merging traditionally separate academic units. Collaboration among professional schools and across the university campus can enhance recruitment efforts by opening new opportunities to develop innovative interdisciplinary educational, research, and clinical programs.

For many, if not most, the appeal of joining an AHS is the ability to create new knowledge, to train the next generation of practitioners, and to develop new processes and systems. Therefore, in faculty recruitment, it is crucial that any clinical duties align with the candidate's teaching and scholarly pursuits. Meanwhile, experienced leaders seeking fresh opportunities are often drawn by the chance to implement transformative changes in the AHS, driven by market forces, new resources, and emerging institutional partnerships. It behooves institutions to recruit and support such leaders.

## Conclusions

Recruitment to AHS has become extremely challenging at all levels as resources become scarcer and qualified talent becomes harder to find. In addition, there may be growing reluctance to relocate amid growing uncertainty. Organizations are only as successful as their people, and finding top talent should be a key priority. Accordingly, recruitment processes must be streamlined and simplified, with the capacity to adapt to changing conditions.

## Funding

This research received no specific grant from any funding agency in the public, commercial, or not-for-profit sectors.

## Declaration of competing interest

DNB and JDH declare that they have no known competing financial or personal relationships that could have appeared to influence the work in this paper. SH discloses that she is principal in Shelli Herman Associates, a search firm for executive positions.
